# Antimicrobial control and temporal dynamics of *M. plutonius* colonization in adult worker honey bees (*Apis mellifera*)

**DOI:** 10.1371/journal.pone.0322770

**Published:** 2025-05-12

**Authors:** Midhun Sebastian Jose, Marina Carla Bezerra da Silva, Oleksii Obshta, Fatima Masood, Jenna M. Thebeau, Sarah Biganski, Muhamad Fahim Raza, Marcelo Polizel Camill, E.E. Tellarini Prieto, Thanuri Edirithilake, Ivanna Kozii, Igor Moshynskyy, Elemir Simko, Sarah C. Wood

**Affiliations:** 1 Department of Veterinary Pathology, University of Saskatchewan, Saskatoon, Saskatchewan, Canada; 2 Department of Veterinary Microbiology, University of Saskatchewan College of Veterinary Medicine, Saskatoon, Saskatchewan, Canada; 3 Prairie Diagnostic Services Inc., University of Saskatchewan, Saskatoon, Canada; Institute of Apicultural Research, CHINA

## Abstract

European foulbrood (EFB) is a stress-associated brood disease affecting honey bee larvae, caused by infection with *Melissococcus plutonius*. Adult bees are suggested to be a reservoir for this bacterium; however, the duration of *M. plutonius* colonization of adult bees and its impact on adult bee survival remain inadequately understood. In North America, the only approved antimicrobial for treatment of EFB is oxytetracycline; yet the antimicrobials tylosin and lincomycin are also widely used in beekeeping. The effect of these antimicrobials on *M. plutonius* colonization of adult bees is unclear. To investigate these unknowns, we infected summer and winter adult worker bees with *M. plutonius* in the laboratory and treated the infected workers with one of three used antibiotics in beekeeping, in either the laboratory, or within field colonies. We found that worker bees remained persistently infected with *M. plutonius* for at least 22–38 days in the laboratory and at least 24 days in field colonies. Moreover, *M. plutonius* ingestion was associated with a significant, dose-dependent decrease in adult worker survival. Antibiotic treatment significantly decreased the *M. plutonius* load of colonized adult workers but failed to eliminate *M. plutonius*; still antibiotic treatment improved the survival of bees in the laboratory cages. Taken together, these findings indicate the importance of adult bees as a reservoir of *M. plutonius*, even in colonies treated with antimicrobials.

## 1. Introduction

European foulbrood (EFB) is a bacterial brood disease of honey bees, caused by larval infection with the bacterium *Melissococcus plutonius*. Larvae are proposed to acquire the pathogen through brood food provided by nursing adult bees, although the dynamics of *M. plutonius* colonization in adult bees is poorly understood, [1,2] Importantly, even adult bees from clinically healthy colonies can harbor significant levels of *M. plutonius* [3], implicating adult worker bees as a reservoir for *M. plutonius.* Moreover, the different genetic variants, or clonal complexes (CCs), of *M. plutonius*, including atypical (CC12) and typical (CC13) variants, have shown variable pathogenicity to honey bee larvae *in vitro* [4–8], but the impact of these variants in adult bees has not been described. Considering EFB is an unpredictable, stress-associated disease, it is important to understand how long adult bees remain persistently colonized with living *M. plutonius* and the impact of this colonization on adult bee survival, to effectively develop integrated pest management (IPM) strategies for EFB.

Currently, IPM for EFB includes regular brood frame replacement, shook swarm (shaking the bees into clean beekeeping equipment), queen caging, and, in severe cases, destroying infected colonies and equipment by incineration [9,10]. In addition, beekeepers in North America, New Zealand and Australia use oxytetracycline (OTC), the only approved antibiotic, for the treatment of EFB. The use of any antibiotics for honey bee colonies is generally not permitted in Europe [11]. Nevertheless, Budge et al. [12] documented the presence of *M. plutonius* in adult bees from colonies routinely treated with OTC in England and Wales, suggesting that adult bees may continue to harbor *M. plutonius* despite antimicrobial therapy. In addition to OTC, the antimicrobials tylosin (TYL) and lincomycin (LMC) are widely used by North American beekeepers for controlling American foulbrood disease. However, use of TYL and LMC for EFB disease treatment is not permitted in bee keeping. Both TYL and LMC have been previously shown to be an effective treatment for honey bee larvae infected with *M. plutonius in vitro* [13]; however, their effectiveness in adult bees colonized with *M. plutonius* has not been explored.

Considering that adult bees can be carriers of EFB, we aimed to determine how long adult bees remain persistently colonized with *M. plutonius*, by performing experimental inoculation of adult bees with *M. plutonius*. Using this experimental approach, we investigated 1) the duration of *M. plutonius* colonization in summer and winter adult worker bees 2) the impact of *M. plutonius* colonization on summer and winter adult worker bee survival 3) and the effect of antimicrobial therapy on the duration of *M. plutonius* colonization. Taken together, the results of these experiments will improve understanding of the role of adult workers as a reservoir for *M. plutonius* which, in turn, will inform management strategies for EFB prevention and control.

## 2. Materials and methods

### 2.1. Materials

#### 2.1.1. Antibiotics.

We tested three antibiotics, including oxytetracycline hydrochloride, tylosin tartrate and lincomycin hydrochloride. Field colonies were treated with formulated oxytetracycline hydrochloride [Oxy tetra-A (55mg/g Oxytetracycline hydrochloride, Dominion Veterinary Laboratories Ltd., Canada. Code: ADS168.20, Lot: Din. 00546704]. Bees in laboratory cages were treated with laboratory-grade oxytetracycline hydrochloride (Fisher Scientific, Canada. Code: J62489.14, lot: U23H060), tylosin tartrate (Fisher scientific, Canada. Code:463070050, lot:AO410945), or lincomycin hydrochloride (Fisher scientific, Canada. Code: J612510.6, Lot: Z28G024).

#### 2.1.2. Experimental bacterial strains.

We evaluated two stains of *M. plutonius* including the CC12 strain 2019BC1, isolated from a clinical case of EFB [13] and the CC13 strain ATCC 35311 (Cedarlane, Ontario, Canada).

#### 2.1.3. Culture media and conditions.

*M. plutonius* 2019BC1 was cultured in KSBHI liquid media (brain heart infusion supplemented with 0.15 M KH_2_PO_4_, and 1% soluble starch [3]) or KSBHI agar media (KSBHI broth with 15 g/L bacteriological agar) at 37°C for 72 h under microaerophilic conditions (Aneropack-MicroAero, MGC, Mitsubishi, Japan) with shaking at 150 rpm for broth media.

*M. plutonius* ATCC 35311 was cultured in basal liquid media (yeast extract supplemented with 0.15 M KH_2_PO_4_, 1% soluble starch, 1% glucose, and 0.025% L-Cysteine) [14]), or KSBHI agar media (Basal broth with 15 g/L bacteriological agar) at 35°C for 72 h under microaerophilic conditions (Aneropack-MicroAero, MGC, Mitsubishi, Japan) with shaking at 150 rpm for broth media.

### 2.2. Experimental animals

#### 2.2.1. Naturally infected summer adult worker bees from a colony with clinical EFB.

Three brood frames containing capped and open brood sourced from a single colony in Saskatchewan, Canada demonstrating clinical signs of EFB were submitted to the University of Saskatchewan in July 2022.

#### 2.2.2. Summer worker bees.

Three brood frames with age-synchronized adult honey bee workers in July 2022 were obtained by caging the queens in three hives for 24 hours with an empty frame of foundation drawn with wax. Frames with eggs were removed from the queen’s cage in each colony and incubated in an adjacent brood chamber of the same colony for 19 days. Next, the frames were transferred to a laboratory incubator at 33°C and 60% relative humidity (RH) until adult bee emergence. All colonies were maintained at a research apiary located at the University of Saskatchewan (Saskatoon, Saskatchewan, Canada) and the colonies were never diagnosed with EFB in the past. The colonies were managed according to the Saskatchewan Apiaries Act [15].

#### 2.2.3. Winter worker bees.

Winter worker bees were collected into laboratory cages using a vacuum in November 2022 and 2023 from the brood chambers of three colonies maintained in an indoor overwintering facility at the University of Saskatchewan. The cages were transported to the laboratory in a portable incubator at 33°C where they were immediately transferred to a laboratory incubator at 33°C and 60% RH.

### 2.3. Methods

#### 2.3.1. Study design.

**Fig 1:**
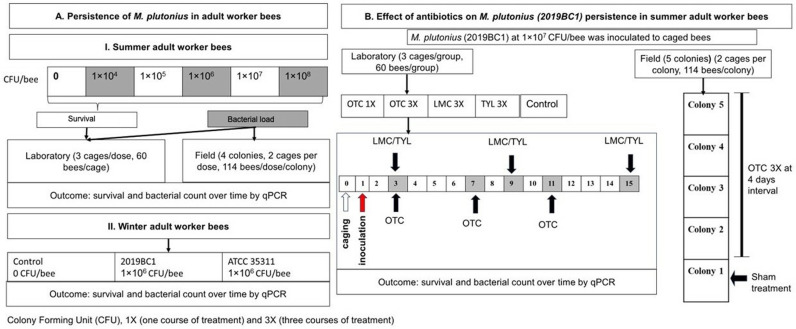
Study design to investigate *M. plutonius* colonization in adult worker bees. **A.** Adult summer and winter worker bees in cages were inoculated with *M. plutonius* and worker survival and *M. plutonius* colonization were monitored. **B.** Effect of antibiotic treatment on the persistence of *M. plutonius* in summer workers treated with antibiotics including OTC (oxytetracycline), TYL (tylosin) and LMC (lincomycin) in cages or OTC in field colonies (Study with OTC-treated colonies was conducted in July, 2023, with sham treatment occurring in June, 2023).

We conducted both laboratory and field studies to describe the dynamics of *M. plutonius* colonization in adult worker bees (Fig 1). First, to determine natural levels of *M. plutonius* colonization in adult bees, we collected newly emerged worker bees from colonies with clinical signs of EFB and performed real-time quantitative PCR (qPCR) analysis to determine the *M. plutonius* bacterial load. Second, we performed experimental inoculation of adult bees with incremental doses of *M. plutonius*, ranging from 1 × 10^4^ to 1 × 10^8^ CFU/bee, and determined the effect on summer adult bee survival in laboratory cages. We repeated these experiments to assess the persistence of *M. plutonius* colonization in summer adult bees in both the laboratory and in field colonies.

Third, we compared the duration of *M. plutonius* colonization in winter adult worker bees using two *M. plutonius* isolates: ATCC 3511 and 2019BC. Finally, to explore the impact of antimicrobial treatment on *M. plutonius* colonization of summer adult worker bees, we performed experimental inoculation of newly emerged adult bees with 1 × 10^7^ CFU/bee *M. plutonius* 2019BC1 in the laboratory and subsequently treated the bees with either (1) oxytetracycline hydrochloride (OTC), tylosin tartrate (TYL) or lincomycin hydrochloride (LMC) in laboratory cages or (2) label doses of OTC applied after introduction of the marked bees into field colonies.

#### 2.3.2. Bacterial inoculum preparation.

The bacterial cultures were grown in liquid media (see 2.1.3). Cells were harvested by centrifugation at 4000 rpm for 20 minutes at 4°C. The pellets were resuspended in 1X phosphate buffered saline (PBS), followed by 10-fold serial dilution in PBS. The bacterial concentration was quantified using both qPCR and standard bacterial plate count. The bacterial concentration was adjusted based on the viable count to the desired levels in 3 mL of sterile sugar syrup [1:1 (w/v)]. The inoculum was given to the caged bees immediately after mixing *M. plutonius* with sugar syrup. The sugar syrup inoculated with the bacteria was also re-plated to confirm the accuracy of the infectious dose administered (S1 table).

#### 2.3.3. Processing of adult worker bees for bacterial culture and DNA isolation.

Adult bees were individually processed by surface sterilization (70% ethanol and then 0.05% sodium hypochlorite), homogenized in 1mL PBS per bee using sterile pestles, and centrifuged for five minutes at 2000 rpm to remove debris. The supernatant was divided into two 500 µ L aliquots, for bacterial culture and DNA extraction, respectively, and stored at ^-^80°C. An equal volume 500 µ L of glycerol was added to the aliquot for bacterial culture to preserve cell viability.

2.3.3.1 *M. plutonius* culture of adult worker bees: To verify if *M. plutonius* CFU detected by qPCR were viable, aliquots of bee homogenate were thawed at room temperature and 100 µ L from three bee homogenate samples were pooled. This sample was then serially diluted and plated in three replicate KSBHI or Basal media agar plates and incubated at 35°C under microaerophilic conditions as described in section 2.2.3. Up to three CFU per plate with colony morphology resembling *M. plutonius*, were sent for identification using Matrix-Assisted Laser Desorption/Ionization Time-of-Flight Mass Spectrometry (MALDI-ToF) (Prairie Diagnostic Services Inc., Saskatoon, SK).

2.3.3.2 DNA isolation from adult worker bees: DNA isolation was performed on either individual bee homogenate (see section 2.3.3) or pooled samples of individual bee homogenates. To generate pooled samples, 100 µ L from each of three samples of individual bee homogenate (see section 2.3.3) were mixed. 100 µ L from the pooled or individual sample was used for DNA isolation with a DNeasy Blood and Tissue Kit (QIAGEN, Germany). The DNA was eluted in 50 µ L nuclease-free water and 2 µ L of this DNA was used for qPCR detection of *M. plutonius* using the assay conditions described by Budge et. al [12] and the corresponding 16s-rRNA based primers and probes: *EFBFor* (TGT TGT TAG AGA AGA ATA GGG GAA), *EFBRev2* (CGT GGC TTT CTG GTT AGA) and *EFBProbe* (FAM - AGA GTA ACT GTT TTC CTC GTG ACG GT - TAMRA)*.* Real-time reactions were performed in triplicates using TaqMan chemistry and Multiplex qPCR reagent kits (QIAGEN, Germany). *M. plutonius* DNA was quantified by comparing it to a standard curve [16]. To prepare the standard curve, DNA was extracted from a spiked sample containing known CFU of *M. plutonius* and a background of *M. plutonius*-free, adult bee homogenate. qPCR was performed on extracted DNA and the Cycle threshold (Ct) values were plotted against the logarithm of the CFU/mL.

#### 2.3.4. Determination of *M. plutonius* load in naturally infected summer adult worker bees from a colony with clinical EFB.

To confirm diagnosis of EFB for the naturally infected brood frames submitted to our laboratory (see section 2.2.1), eight larvae from each frame were individually sampled using sterile swabs and processed for qPCR (see section 2.3.3.2). The frames were immediately transferred to an incubator at 33°C and 60% RH to allow adult bees to emerge from the capped brood on each frame. To determine *M. plutonius* load of adult workers, 12 newly emerged adult bees were sampled from each of the four frames (three bees per frame were pooled for DNA extraction, n = 12) for *M. plutonius* quantification by qPCR.

#### 2.3.5. Detection of *M. plutonius* colonization retention rate in summer adult worker bees in the laboratory.

2.3.5.1 Adult summer bee survival: To evaluate the effect of *M. plutonius* colonization on adult summer bee survival in the laboratory, newly-emerged worker bees from three brood frames (representing three queens or genetic lines) were caged in stainless steel insect cages (each cage measured 8.5 x 5 x 9 cm; 60 bees per cage) 12–24 hours after emergence [experimental day 0 (D0)], provided with *ad libitum* 1:1 (w:v) sterile sugar syrup, and maintained at 33°C and 60% RH. An additional 12 bees were sampled on D0 for DNA isolation and qPCR to verify the absence of *M. plutonius* prior to experimental infection. On D1, each cage was provided with a syringe feeder containing one of six incremental doses of 2019BC1 *M. plutonius* (three cages per dose) including 0, 1 × 10^6^, 1 × 10^7^, 1 × 10^8^, 1 × 10^9^, and 1 × 10^10^ CFU *M. plutonius* in 3 mL of 1:1 sterile sugar syrup, with calculated doses of 0, 1 × 10^4^, 1 × 10^5^, 1 × 10^6^, 1 × 10^7^, 1 × 10^8^ CFU/bee, respectively, based on 60 bees per cage. *M. plutonius* inoculum was fully consumed in 24 hours (D2), and each cage was provided with sterile 1:1 sugar syrup *ad libitum* thereafter, with consumption recorded at 24-hour intervals. Adult bee survival was monitored every 24 hours and dead bees were removed. Afterwards, 3 bees per cage were sampled for qPCR on D3 to confirm *M. plutonius* colonization. The experiment was terminated at D22 when there were no bees remaining in the cages.

2.3.5.2 Duration of *M. plutonius* colonization: To evaluate the duration of *M. plutonius* colonization of adult bees in the laboratory, the above experiment was repeated using four infectious doses of *M. plutonius* 2019BC1 selected from the previous experiment, including control (0 CFU/bee), low (1 × 10^4^ CFU/bee), medium (1 × 10^6^ CFU/bee), and high (1 × 10^8^ CFU/bee) doses with three cages of 60 bees per dose. Consumption and survival were monitored at 24-hour intervals. Beginning on D3 and continuing at four-day intervals until D22, three bees per cage (nine bees per dose) were collected and processed individually for the determination of *M. plutonius* infectious load by qPCR. To verify if *M. plutonius* detected by qPCR was viable, bacterial culture for *M. plutonius* was performed on pooled samples of 3 bees sampled on D3 and D22 (see section 2.3.3.1).

#### 2.3.6. Persistent detection of *M. plutonius* colonization of summer adult worker bees in a colony.

Similar to the laboratory experiments described in section 2.3.5.2, newly emerged adult bees were caged on D0 and infected on D1 with incremental doses of *M. plutonius* 2019BC1 [0 CFU/bee, low (1 × 10^4^ CFU/bee), medium (1 × 10^6^ CFU/bee), and high (1 × 10^8^ CFU/bee) dose] with 60 bees per cage, and eight cages per dose. Before caging, the bees were paint-marked on the thorax with a water-based queen marker (POSCA, Japan) with different colors corresponding to their treatment group. The cages were maintained in a laboratory incubator at 33°C and 60% RH for 24 hours until consumption of the *M. plutonius* inoculum was complete. Subsequently, bees were transferred to the field on D2, where the caged bees were provided *ad libitum* 1:1 w/v sugar syrup for 24 hours within an empty super (box) placed on top of each of four, two-brood-chamber colonies (2 cages per dose per colony = 2 cages x 4 dose groups x 60 bees/cage = 480 bees/colony) separated by queen excluder to allow for pheromone exchange before the release of the experimental bees from the cages into the colony on D3. Before releasing into the colony, three bees from each cage (24 bees per treatment) were collected on D3 to confirm *M. plutonius* colonization by qPCR of individual bees. After the introduction to the colonies on D3, 12 marked bees per treatment per colony were collected at four-day intervals until D24, after which no more marked bees could be recovered from the colonies. *M. plutonius* infectious load by qPCR was determined on individual bee samples on D3 and, thereafter, on pooled samples of 3 bees. To verify if *M. plutonius* detected by qPCR was viable, bacterial culture for *M. plutonius* and MALDI-TOF identification was performed on pooled samples of three bees sampled on D3 and D24.

#### 2.3.7. Persistent detection of *M. plutonius* colonization of adult winter worker bees in the laboratory.

Adult winter worker bees (60 bees per cage) in laboratory cages kept at 33°C and 60% RH were provided with 1:1 (w:v) sugar syrup ad libitum through syringe feeders. After a 12-hour acclimatization, the cages were provided with control 1:1 sugar syrup (3 cages) or 1:1 syrup containing 1 × 10^6^ CFU/bee *M. plutonius* [ATCC 35311 (3 cages) or *M. plutonius* 2019BC1 (3 cages)]. *M. plutonius* inoculum was fully consumed in 24 hours (D2), and each cage was provided with sterile 1:1 sugar syrup ad libitum thereafter, with consumption recorded at 24-hour intervals. The survival of bees in the cages was also recorded daily. Three bees per cage (nine bees per group) were collected for the determination of *M. plutonius* infectious load by qPCR on D0 (pre-inoculation), D3, D8, D22, D26, D30, D34 and D38, until no bees remained in the cages. *M. plutonius* infectious load by qPCR was determined on pooled samples of 3 bees. To verify if *M. plutonius* detected by qPCR was viable, bacterial culture for *M. plutonius* was performed on pooled samples of 3 bees sampled from each cage on D3 and D38.

#### 2.3.8. Persistent detection of *M. plutonius* colonization of summer adult worker bees treated with antimicrobials in the laboratory.

Similar to the experiments described in sections 2.3.5.2, newly emerged adult worker bees were caged (60 bees/cage) on D0 and maintained at 33ºC and 60% RH. On D1, 18 cages received control syrup, and 18 cages received syrup containing 1 × 10^7^ CFU/bee *M. plutonius* 2019BC1. Once the inoculum was completely consumed on D3, three bees from each cage were sampled for determination of *M. plutonius* bacterial load by qPCR and bacterial culture to confirm establishment of bacterial colonization. Next, the cages were fasted for 4 h and subsequently each fed with 4mL of control, 1:1 w/v sugar syrup (6 cages) or 4mL of 1:1 w/v sugar syrup containing one of three antibiotics (18 cages) at label doses [0.1mg/mL TYL three times (3X), 0.05mg/mL LMC three times (3X), 0.1mg/mL OTC three times (3X) or 0.1mg/mL OTC one time (1X)]. We followed the label dosage of antibiotics and intervals, with 4–5-days apart for OTC as per the label treatment and one week apart as per the label for TYL/LMC treatment. Each treatment group had 6 cages and each cage with 60 bees in it. Three applications of OTC treatment was done at 4 days interval and 3 application of TYL and 3 applications of LMC treatment was performed at 6 day intervals. All cages received ad libitum control 1:1 w/v sugar syrup in between treatments. On D3 (pre-treatment), and post-treatment, on D15 (4 days post treatment) for the OTC group or D21 (6 days post treatment) for the TYL and LMC groups, three bees per cage (nine bees per treatment group) were sampled for the determination of *M. plutonius* infectious load using qPCR. Verification of *M. plutonius* viability using bacterial culture on a pooled sample of three bees was done on D3, D15 (OTC treatment group) and D21(LMC/TYL treatment group).

#### 2.3.9. Persistent detection of *M. plutonius* colonization of summer adult worker bees treated with OTC in a colony.

In July 2023, similar to the field experiment described in section 2.3.5, newly emerged summer adult worker bees were paint-marked and caged (60 bees/cage) on D0. On D1, the caged bees were provided with 1:1 w/v sugar syrup containing 0 CFU/bee or 1 × 10^7^ CFU/bee *M. plutonius* 2019BC1, with eight cages per group. To confirm *M. plutonius* colonization, three bees from each cage (24 bees per group) were collected on D3 for qPCR. Next, the bees were released into 4 colonies (2 cages per treatment per colony = 2 cages x 5 colony x 57 bees/cage = 570 bees/colony). To encourage experimental syrup consumption the hives were moved into a 10 ft x 10 ft mesh tent 24-72h before introducing the caged bees, and the colonies were kept inside the tent throughout the experiment. 4 treatment colonies, each received 2L 1:1 w/v sugar syrup (colonies were top fed through a light-proof, 4L glass jar) containing OTC at a concentration of 3.65g/L of formulated product which is equivalent to 200mg of active substance (100mg/L). Syrup consumption was monitored every 24h and the unconsumed volume was replaced with new syrup to avoid degradation of OTC. The colonies were fed ad libitum with control syrup after treatment completion. The colonies were fasted 12h before each treatment. Bees were sampled from each colony (n = 12 bees per colony per sampling point) on D7 (before 2^nd^ treatment), D11 (before 3^rd^ treatment) and D15 (after 3^rd^ treatment) and the *M. plutonius* load was determined by qPCR on pooled samples of 3 bees. To verify if *M. plutonius* detected by qPCR was viable, bacterial culture for *M. plutonius* was performed on pooled samples of 3 bees sampled from each colony on D3 (before treatment) and D15 (after treatment). Due to lack of age-matched, newly emerged adult bees, the control group (2L 1:1 w/v syrup) could not be performed at the same time as the treatment groups. Thus, data from a previous, identical experiment from June 2023 was used. The samples from the control groups were stored at -20ºC and processed together with the samples from the current study.

### 2.4. Ethics statement

This study did not involve the use of endangered or protected species.

### 2.5. Statistical analysis

Statistical analyses were performed using SPSS version 20 (IBM, USA). We considered differences significant at P < 0.05. Data are presented as mean ± standard deviation (SD) for normally distributed data and medians and interquartile range (IQR) for non-normally distributed data. Normality was assessed using the Shapiro-Wilk test and equality of variance was assessed using Levene’s test. Sugar consumption data were analyzed using a two-way repeated measures analysis of variance (ANOVA) with Dunnett’s multiple comparison test. Survival was analyzed using Kaplan-Meier survival analysis and a Mantel-Cox log-rank test*. M. plutonius* bacterial load per bee (CFU/bee) was compared between sampling days using a Kruskal-Wallis test with Dunn’s multiple comparison test.

## 3. Results

### 3.1. Determination of *M. plutonius* load in naturally infected, freshly emerged, summer adult worker bees from a colony with clinical EFB

Newly emerged adult bees (n = 8 pooled samples of 3 bees) from a naturally-infected frame had a mean of 4.3 × 10^7 ^± 5.8× 10^7^ CFU *M. plutonius*/bee (Range = 1.8 × 10^6^ to 1.8 × 10^8^), while sick larvae (n = 8, pooled samples of 3 larvae) from a naturally infected frame carried a mean of 1.4 × 10^9 ^± 1 × 10^9^ CFU *M. plutonius*/larvae (Range = 1.2 × 10^8^ to 2.9 × 10^9^)

### 3.2. Persistent detection of *M. plutonius* in summer adult worker bees in the laboratory

**Fig 2:**
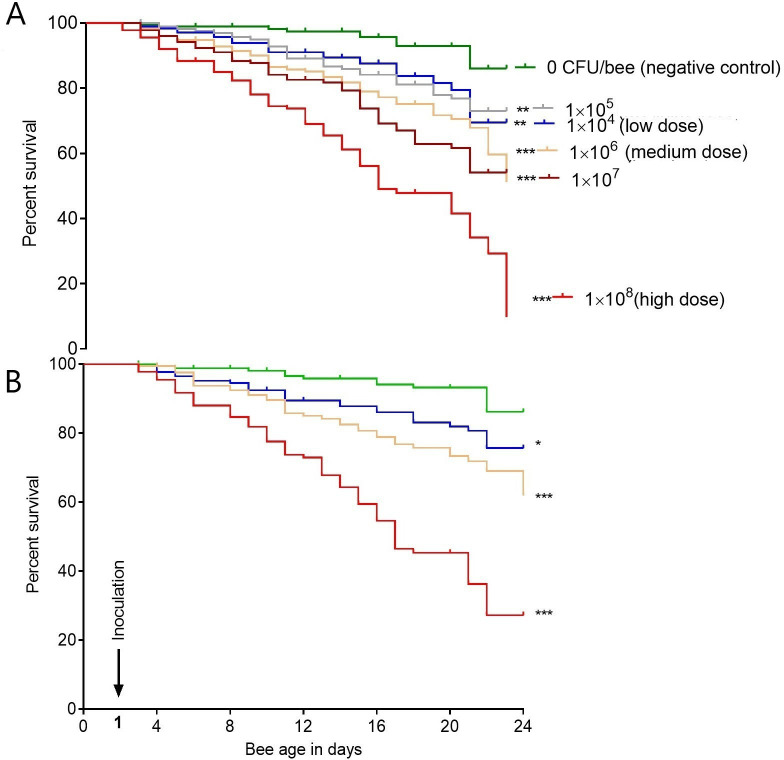
Mean percent survival of adult summer worker bees (n = 180/group) over time (in days) after inoculation with incremental doses of (CFU/bee) of *M. plutonius* in laboratory cages. Figures A and B represent replicate experiments. *,** and *** indicate significant differences from negative control with P < 0.05, P < 0.01 and P < 0.001, respectively.

In both replicate trials (Fig 2A and Fig 2B), *M. plutonius* inoculation significantly (χ ^2^ = 118.9, P < 0.001) decreased adult summer worker bee survival in a dose-responsive manner compared to control over 24 days. In the non-inoculated, negative control group, 86 ± 3.1% of bees survived until 24 days, while in contrast, only between 10% (IQR = 13.2%) (Fig 2A) and 27% (IQR = 1.3%) (Fig 2B) of bees survived to D24 after inoculation with the highest dose of *M. plutonius* (1 × 10^8^ CFU/bee; P < 0.001). Lower doses of *M. plutonius*, from 1 × 10^4^ to 1 × 10^7^ CFU/bee, also resulted in significant decreases in survival compared to negative control group by 17% (IQR = 2.2%) (P < 0.01) to 32% (IQR = 2.2%) (P < 0.001), respectively.

**Fig 3:**
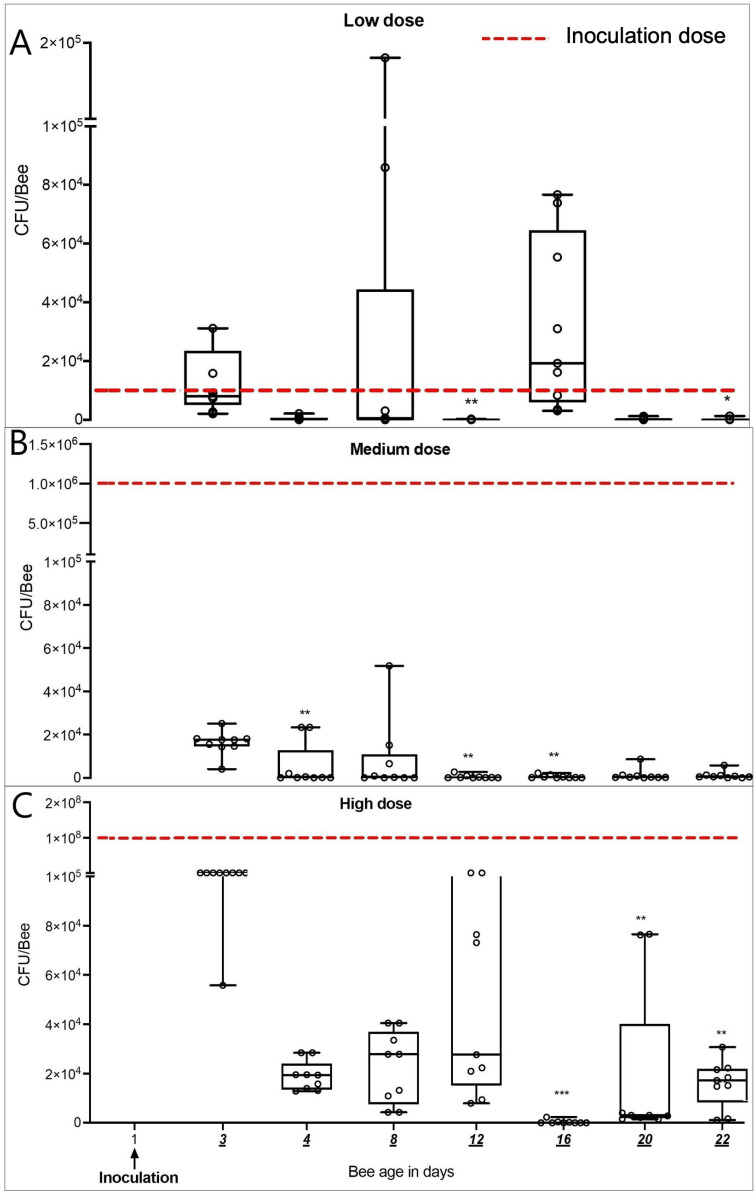
Median ± IQR *M. plutonius* bacterial load (CFU/bee) of caged adult summer worker bees over time after inoculation with A. low (1 × 10^4^ CFU/bee)B. Medium (1 × 10^6^ CFU/bee) and C. high doses (1 × 10^8^ CFU/bee) of *M. plutonius* 2019BC1 on day 1. Each circle represents the bacterial load determined by qPCR in CFU/bee (n = 9 bees per day). *, **, *** indicate significant differences from D3 with P < 0.05, < P < 0.01, and P < 0.001, respectively.

*M. plutonius* was persistently detected in summer adult bees for up to 22 days after inoculation in the laboratory (Fig 3). We observed a significant reduction in bacterial load of inoculated bees over time in all dose groups. *M. plutonius* was not detected by qPCR in control bees nor in bees before inoculation on D0. On day 3 post-inoculation, bees in the low, medium, and high dose groups had reductions of 19.8% (IQR = 86%), 98% (IQR = 0.4%), and 99.8% (IQR = 0.9%) CFU/bee, respectively, relative to the inoculation dose of *M. plutonius* administered on D1 (Fig 3). Relative to D3, *M. plutonius* bacterial load on D22 decreased in all inoculated groups by 99.6% (IQR = 2.4%), 97.1% (IQR = 5.2%), and 88.5% (9.1%) in the low, medium, and high dose groups, respectively, with a median (IQR) *M. plutonius* bacterial load of 33 (186), 497 (926), and 1.7 × 10^4^ (1.3 × 10^4^) CFU/bee on D22 in the low, medium, and high dose groups, respectively. Significant differences between D3 and D22 were only observed in the low (Z = 3, P = 0.02) and high dose groups (Z = 3, P = 0.008). In the low dose group, the median *M. plutonius* load on D16 [1.9 × 10^4^ (5.8 × 10^4^) CFU/bee] was higher than the inoculation dose on D1. Also, in low dose group on D8, some bees (3 samples) had higher *M. plutonius* loads (94% higher) compared to the inoculation dose of 1 × 10^4^ CFU. Bacterial culture on a pooled sample of 3 bees on D3 and D22 were positive for *M. plutonius* based on MALDI-ToF identification (S2 Table).

Consumption of sugar syrup significantly decreased over time (F _110,264_ = 2.0, P < 0.001) in all groups (S1 Fig). The mean sugar consumption per day was between 31.9 ± 3.1 µ L/bee (S1A Fig) to 31.6 ± 5.5 µ L/bee (S1B Fig). Overall, consumption of groups inoculated with *M. plutonius* was similar to control, with significant decreases, from 19.2%-78.2%, in consumption of *M. plutonius* inoculated groups relative to control, on days 15, 18, and 19 (S1A Fig) in the first experimental trial and on days 8, 12, 14, and 17 in the second, replicate experiment (S1B Fig).

### 3.3. Detection of *M. plutonius* colonization in summer adult worker bees in a colony

**Fig 4:**
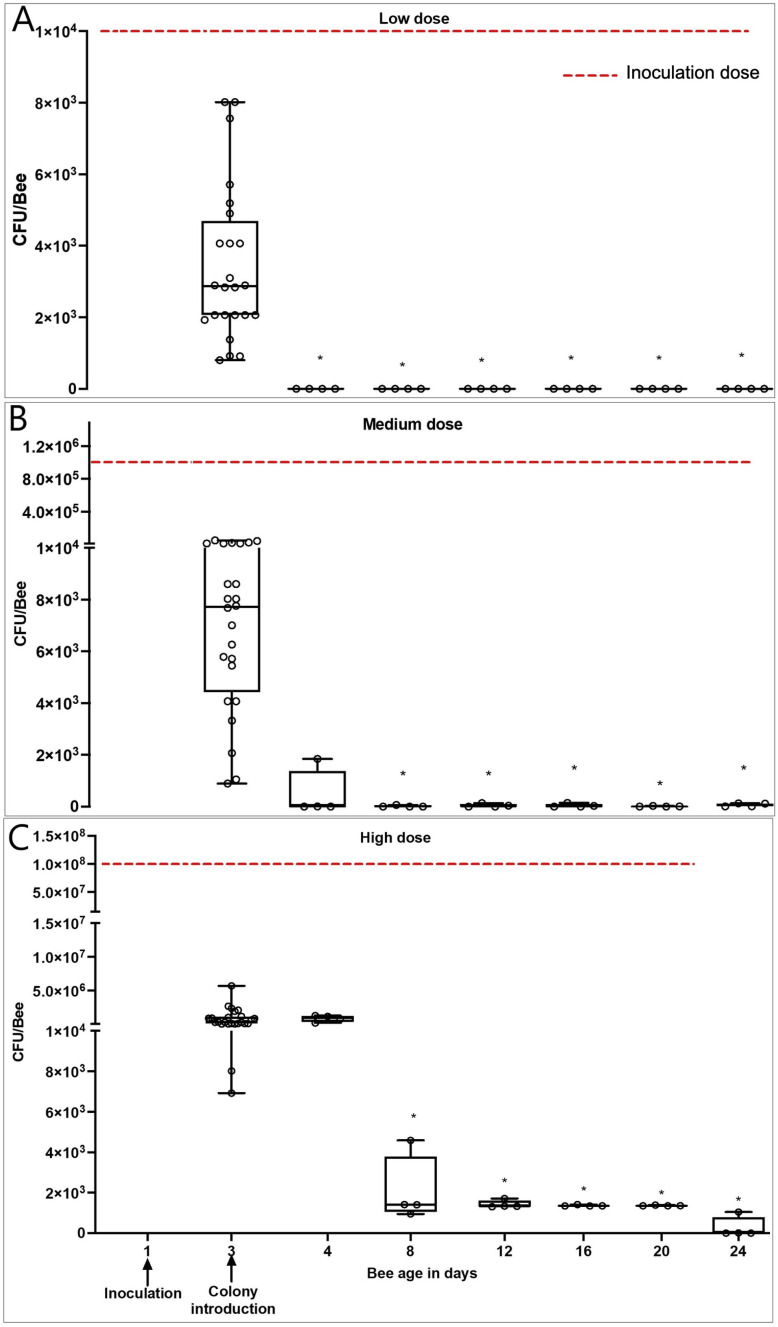
Median ± IQR *M. plutonius* bacterial load (CFU/bee) of adult summer worker bees over time after introduction on D3 into field colonies. A, B, and C represent bees inoculated on D1 with low (1 × 10^4^ CFU/bee) medium (1 × 10^6^) and high doses (1 × 10^8^ CFU/bee) of *M. plutonius* 2019BC1. Each circle represents the bacterial load determined by qPCR in CFU/bee [(each dot on D3 represents 1 bee with n = 24 and dots on days 4 to 24 each represent 3, pooled bees (n = 4 pooled samples/day)]. * Indicates significant differences from D3 with P < 0.05.

*M. plutonius* 2019BC1 was persistently detected in experimental summer adult worker bees for up to 24 days within field colonies (Fig 4) in the bees inoculated with medium (1 × 10^6^ CFU/bee) and high (1 × 10^8^ CFU/bee) doses of *M. plutonius*, carrying a mean of 59.5 ± 32.2 and 267.1 ± 259.6 CFU/bee on D24 in the medium and high dose groups, respectively. However, in the low dose group, *M. plutonius* was not detected in any of the inoculated bees following colony introduction from D4 to D24. *M. plutonius* was not detected by qPCR in control bees nor in bees before *M. plutonius* inoculation. On day 3 post-inoculation, bees in the low, medium, and high dose groups had 71.3% (IQR = 26.3%), 99.2% (IQR = 0.7%), and 99.6% (IQR = 1.1%) less CFU/bee, respectively, relative to the inoculation dose of *M. plutonius* administered on D1 (Fig 4). Relative to D3, *M. plutonius* bacterial load on D24 decreased in all inoculated groups by 100%, 99.2%, and 99.9% in the low, medium, and high dose groups respectively. Significant differences between D3 and D24 were observed in the low (Z = 2.8, P = 0.0008), medium (Z = 2.8, P = 0.02) and high dose (Z = 4.1, P = 0.012) groups. Bacterial culture on a pooled sample of 3 bees on D3 and D24 were positive for *M. plutonius* based on MALDI-ToF identification (S3 Table).

### 3.4. Persistent detection of *M. plutonius* in adult winter worker bees in the laboratory

**Fig 5:**
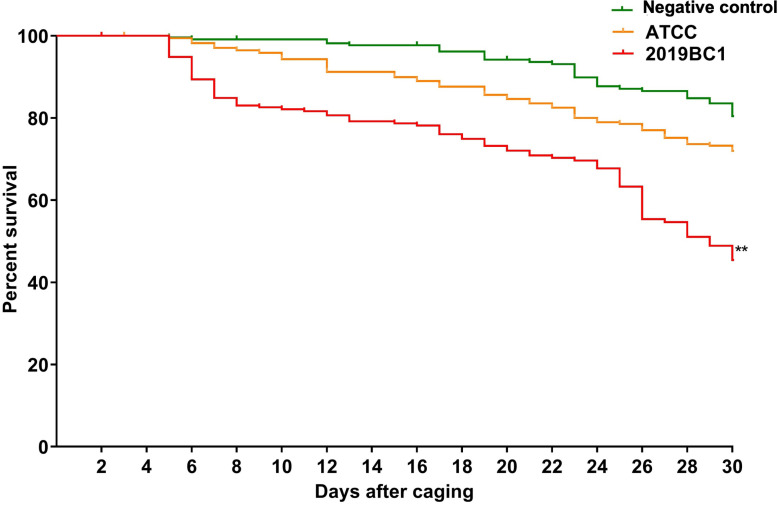
Mean percent survival of adult winter bees (n = 180/group) over time (in days) after inoculation with 1 × 10^6^ CFU/bee *M. plutonius* ATCC3511 or 2019BC1 in laboratory cages. ** represents significant differences from negative control at D30 with P < 0.01.

*M. plutonius* 2019BC1 inoculation significantly decreased adult winter bee survival by 56% (IQR = 24.8%) after 30 days, relative to non-inoculated, control bees (χ2 = 55.4, P < 0.001); however, inoculation of winter bees with *M. plutonius* ATCC 35311 did not significantly affect the survival compared to the non-inoculated control (Fig 5).

**Fig 6:**
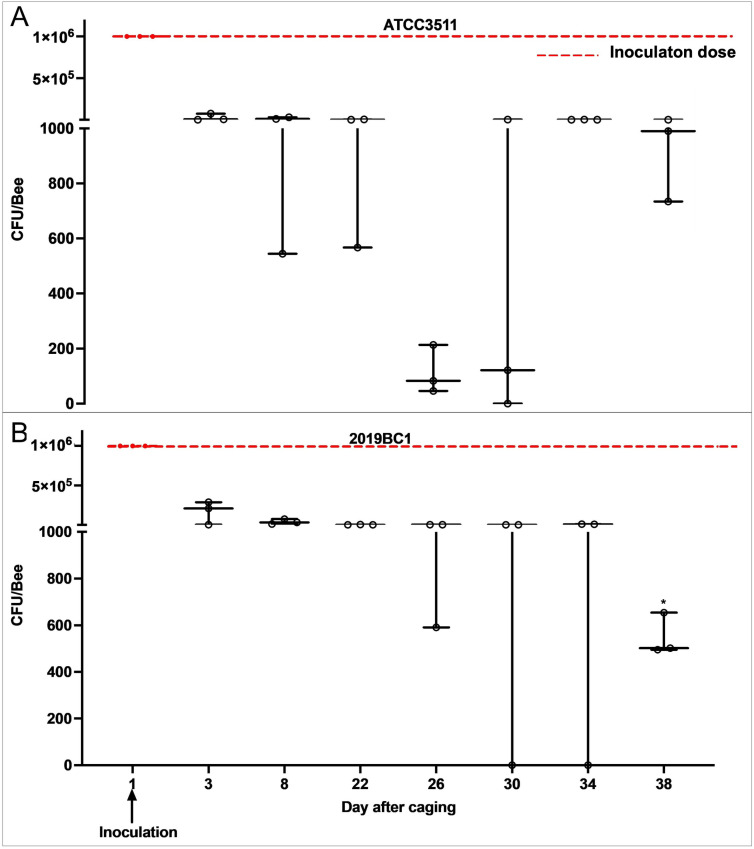
Median ± IQR *M. plutonius* bacterial load (CFU/bee) of adult winter worker bees in the laboratory inoculated with A. *M. plutonius* ATCC3511 or B. *M. plutonius* 2019BC1. Each circle represents the bacterial load in CFU/bee determined by qPCR. Each circle represents 3 pooled bees (n = 3 pooled samples/day). Red-dotted line represents calculated inoculation dose (CFU/bee) administered on day 1. * Represents significant differences from D3 with P < 0.05.

Winter adult worker bees remained persistently colonized with either *M. plutonius* ATCC 35311 or 2019BC1 for up to 38 days in laboratory cages carrying between 990 ± 550 and 550 ± 51.9 CFU/bee on D38 in the ATCC35311 and 2019BC1-inoculated groups, respectively (Fig 6). *M. plutonius* was not detected by qPCR in control bees nor in bees before *M. plutonius* inoculation. On day 3 post-inoculation, bees in the ATCC3511 and 2019BC1-inoculated groups had 97.2% (IQR = 95.9%, 2.7 × 10^4^ ± 2.4 × 10^4^ CFU/bee) and 83.3% (IQR = 85.3%, 1.7 × 10^5^ ± 8.4 × 10^4^ CFU/bee) reduction of CFU/bee, respectively, relative to the inoculation dose of 1 × 10^6^ CFU *M. plutonius* administered on D1 (Fig. 6). However, a statistically significant reduction in bacterial load was observed only on day 38 in bees inoculated with 2019BC1 (P = 0.04). Bacterial culture on a pooled sample of 3 bees on D3, and D38 were positive for *M. plutonius* based on MALDI-ToF identification (S6 Table).

Syrup consumption significantly decreased over time (F_2.8, 24.8_ = 17.9, P < 0.001) in all experimental groups (S3 Fig). The mean sugar consumption per day was 42.4 ± 11.1 µ L/bee. Syrup consumption was similar across all groups; however, only the 2019BC1-inoculated group consumed significantly less (by 8–12%) syrup than negative control on days 3 (P = 0.02), 4 (P = 0.03), and 30 (P < 0.001).

### 3.5. Persistent detection of *M. plutonius* colonization of summer adult worker bees treated with antimicrobials in the laboratory

**Fig 7:**
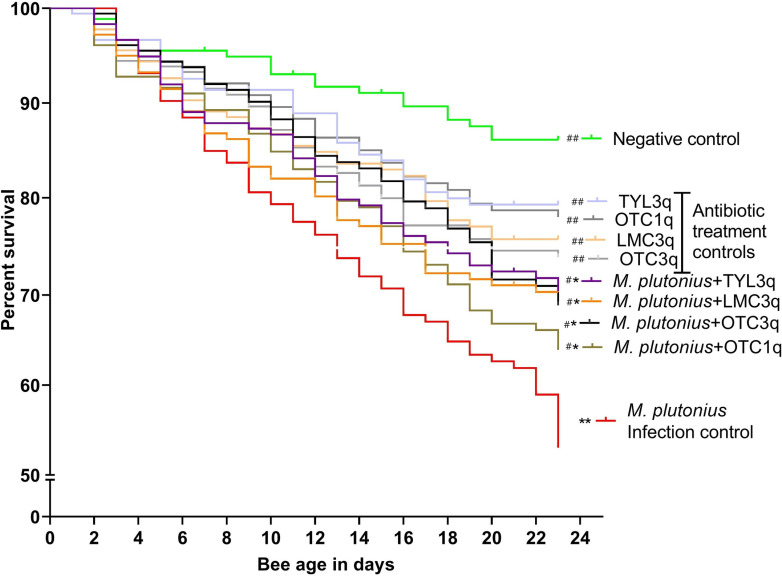
Mean percent survival of newly emerged worker bees (n = 180/group) in the laboratory inoculated with 1 × 10^7^ CFU/bee *M. plutonius* 2019BC1 and/or one (1X) or three (3X) treatment of oxytetracycline (OTC) lincomycin (LMC) or tylosin (TYL). ^#^, ^##^ represents significant differences from *M. plutonius *infection control (inoculated, sham treatment) with P < 0.05 and P < 0.01 respectively, and *,** represents significant differences from negative control (non-inoculated, sham treatment) with P < 0.05 and P < 0.01 respectively.

Antibiotic treatment of adult bees with OTC, TYL, or LMC was associated with a significant, on average, 22% (IQR = 1.8%, χ^2 ^= 12.2, P = 0.02) increase in survival after inoculation with *M. plutonius* compared to non-treated bees inoculated with *M. plutonius* (Fig 7). However, survival of the antibiotic-treated, *M. plutonius*-inoculated bees remained significantly lower, by 21% (IQR = 1.4%) on average, (χ^2 ^= 19.7, P < 0.001) than the negative control, non-inoculated bees. In the absence of *M. plutonius* inoculation, bees treated with antibiotics did not have significantly different survival from the non-treated, negative control bees. For bees inoculated with *M. plutonius*, there were no significant differences in survival among the antibiotic-treated groups (χ^2 ^= 0.9, P = 0.3). Moreover, one treatment with OTC was not associated with a significant difference (χ^2^ = 1.1, P = 0.3) in survival relative to three treatments with OTC.

**Fig 8:**
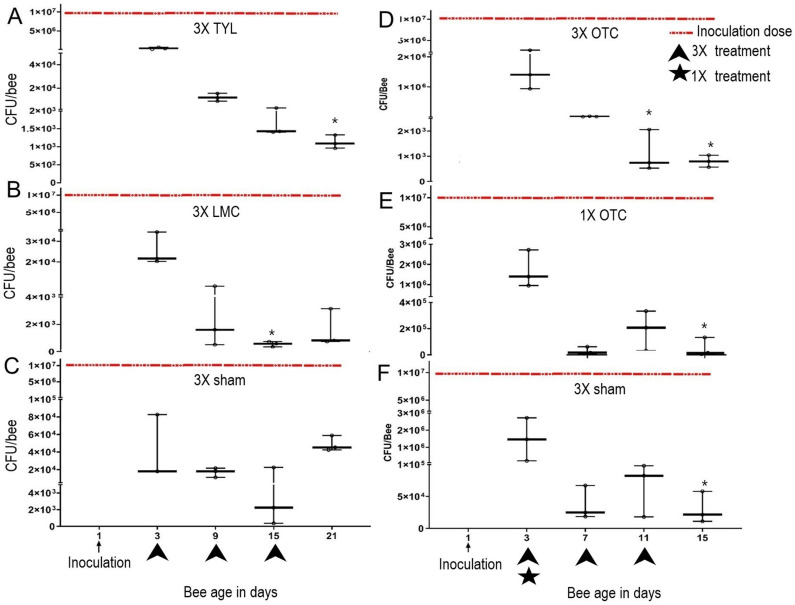
Median ± IQR *M. plutonius* bacterial load (CFU/bee) of newly emerged summer worker bees inoculated with 1 × 10^7^ CFU/bee *M. plutonius* 2019BC1 and/or treated withA. TYL (3 times, q7D), B. LMC (3 times, q7D), C. sham (3 times, q7D), D. OTC (3 times, q4D), E. OTC (1 time) and F. sham (3 times, q4D. Red-dotted line represents calculated inoculation dose (CFU/bee) administered on day 1. *, represents significant differences from D3 bacterial load, with P < 0.05.

On D3 post-inoculation, prior to antibiotic or sham treatment, all experimental groups had on average 7.1 × 10^5^ ± 4 × 10^5^ CFU/bee, representing a 71% (IQR = 40%) decrease from the calculated inoculation dose of 1 × 10^7^ (Fig 6). From D3 to D15-21, *M. plutonius* bacterial load decreased significantly over time in both control (by 92.5%) and antibiotic-treated groups (97.9–99.4%; Fig 8). After completion of the course of antibiotic treatment on day 15 (OTC) or 21 (TYL and LMC), for both control and antibiotic-treated bees, *M. plutonius*, was still detectable by qPCR with an average of 4.9 × 10^4^ ± 1.4 × 10^3^ CFU/bee in control and 1.3 × 10^4^ ± 1.8 × 10^4^ CFU/bee in antibiotic-treated bees. Relative to D3, after completion of treatment on D15 or D21, significant differences in bacterial load were observed in the TYL-treated group [99.35% decrease (IQR = 0.1%), P = 0.007; Fig 8A], the 3X OTC-treated group [99.9% decrease (IQR = 0.06%), P = 0.04; Fig 8D] and the 1X OTC-treated group [98.9% decrease (IQR = 9%), P = 0.004; Fig 8E]. Similarly, we observed a significant, 98.5% (IQR = 3%), (P = 0.04; Fig 8F) reduction in *M. plutonius* CFU in the OTC sham-treated, control group on D15; however, we did not see the significant difference in TYL/LMC sham-treated control group on D21 (Fig 8C). For the LMC-treated group, a significant decrease in bacterial load relative to D3 was observed after the 2^nd^ treatment on D15 [97.7% (IQR = 1.7%), P = 0.01, Fig 8B], with a non-significant (P = 0.3) 96.2% (IQR = 11%) decrease in bacterial load after the 3^rd^ treatment on D21. Bacterial culture on a pooled sample of 3 bees on D3, D15 and D21 were positive for *M. plutonius* based on MALDI-ToF identification (S4 Table).

Syrup consumption was similar among antibiotic-treated and control groups (S3 Fig). The mean sugar consumption was 32.66 ± 5.62 µ L/bee/day. Consumption of sugar syrup significantly decreased over time (F_115,276_ = 1.4, P = 0.02) in all groups. Overall, consumption of groups inoculated with *M. plutonius* was similar to control, with significant decreases, from 37%-56%, in consumption of the inoculated, non-treated group and the inoculated antibiotic treated groups relative to the negative control on days 6, 13 and 21.

### 3.6. Persistent detection of *M. plutonius* colonization of summer adult worker bees treated with OTC in a colony

**Fig 9:**
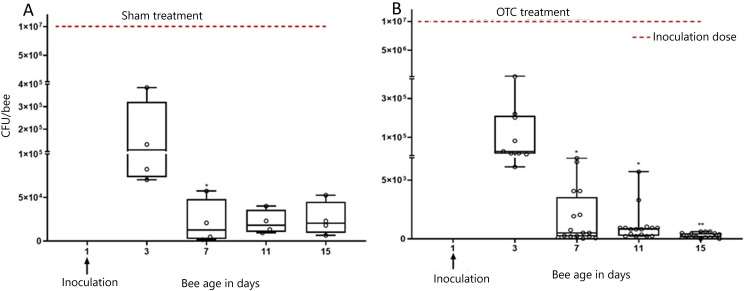
Median ± IQR *M. plutonius* bacterial load (CFU/bee) of caged adult summer worker bees (n = 114 bees/group) introduced on day 3 into field colonies later treated 3 times at 4 days interval with sham or oxytetracycline treatment. **A**. sham treatment, **B.** OTC treatment. Each dot represents the bacterial load determined by qPCR. Each dot on day 3 in sham treatment represents 1 bee per cage (n = 6). Each dot on day 3 in OTC treatment represent 1 bee per cage (n = 18) and each dot on days 4 to 24 represent pooled samples of 3 bees. Red-dotted line represents calculated inoculation dose (CFU/bee) administered on day 1. *,** indicates significant differences from D3 bacterial load with P < 0.05, and P < 0.01 respectively.

Adult worker bees remained persistently colonized with *M. plutonius* after introduction into colonies treated with label-doses of OTC (Fig 9A), carrying on average 444.8 ± 84.8 CFU/bee, one week post treatment on Day 15. *M. plutonius* was not detected by qPCR in control bees nor in bees prior to inoculation. On D3 post-inoculation, prior to antibiotic or sham treatment, the antibiotic-treated and control groups had on average 1.7 × 10^5^ ± 7.4 × 10^4^, and 1.2 × 10^5 ^± 5.5 × 10^4^ CFU/bee, respectively representing a 99.4% (1.9%) and 98.9% (IQR = 2.5%) CFU/bee decrease of *M. plutonius*, respectively, from the inoculation dose of 1 × 10^7^ CFU/bee. From D3 to D15, *M. plutonius* load was significantly decreased in antibiotic-treated colonies [99.5% decrease (IQR = 0.7%), P < 0.001]. However, we observed a significant, 88.2% (IQR = 42.9%; P = 0.02) reduction in *M. plutonius* CFU on D7 in adult bees from the sham treated colony. Additionally, from D11 to D15, *M. plutonius* bacterial load was decreased non-significantly in sham-treated [80.9% decrease (IQR = 33.4%), P = 0.08] colonies. Bacterial culture on a pooled sample of 3 bees on D3, and D15 were positive for *M. plutonius* based on MALDI-ToF identification (S5 Table).

## 4 . Discussion

We investigated persistence of *M. plutonius* colonization in adult bees and the effect of antibiotic treatment on the duration of persistence. We found that adult worker honey bees were detected with *M. plutonius* for at least 22–38 days in laboratory cages and for at least 24 days in field colonies. We report for the first time, a significant decrease in survival of adult bees after *M. plutonius* inoculation in the laboratory. In contrast to our results, Cecchini et al. 2016) [17] demonstrated that inoculation of *M. plutonius* in caged workers did not significantly affect their survival. Additionally, we demonstrated that adult bees remained persistently colonized despite antimicrobial therapy; however, antibiotic treatment improved the survival of bees in cages.

Adult bees are considered carriers of *M. plutonius* [2]; however, the duration of the persistence is unknown. We found that adult bees emerging from naturally infected frames were carrying 4.3 × 10^7 ^± 5.8× 10^5^ CFU/bee, which agrees with the previous studies where adult bees in colonies with clinical symptoms were found to harbor up to 10^7^ CFU/bee [3,18]. Based on these results, we chose a range of experimental inoculation doses of *M. plutonius* which included field-realistic doses. Following inoculation, we determined that summer adult bees carried the bacterium for at least 22 days in laboratory cages and 24 days in field colonies. Regardless of *M. plutonius* inoculation dose (low, medium and high) on D1, we observed a significant, decrease in bacterial load of adult bees on D3 relative to the inoculation dose on D1. Interestingly, in caged workers inoculated with 1 × 10^4^ CFU on D1 in laboratory cages, we found an increase in CFU relative to the inoculation dose on Days 8 and 16, which may indicate the proliferation of bacteria in adult bee gut (Figure 3A). In laboratory cages, bees inoculated with a low dose of *M. plutonius* (1 × 10^4^ CFU) remained persistently colonized until D22, but in field colonies, bees inoculated with a low dose only had detectable *M. plutonius* on D3 post-inoculation, with no *M. plutonius* detectable from D4-24. This discrepancy could be explained by the controlled experimental conditions in the cages versus the constant interaction of bees in the colony with changing microbiome and versatile food sources. [19,20]. Nevertheless, bees inoculated with a field-realistic dose of 1 × 10^6^ CFU *M. plutonius*, still had detectable *M. plutonius* 24 days after inoculation.

Similar to summer bees in laboratory cages, winter bees maintained in the laboratory remained colonized with *M. plutonius* until experiment termination after 38 days. Bacterial load in bees inoculated with ATCC 35311 and 2019BC1 were similar until D34; however, we recorded a significant decrease in *M. plutonius* load on D38 in 2019BC1 inoculated bees. The reduction of CFU/bee on D38 in 2019BC1 could be due to the strain dependent changes in growth dynamics and nutrient utilization. Considering that we found winter bees carried *M. plutonius* for the duration of their lifespan in the laboratory, winter bees in field colonies may represent a reservoir of *M. plutonius* which contributes to occurrence of clinical EFB the following spring. However, contaminated brood comb and beekeeping equipment could also be a potential disease reservoir.

Inoculation of adult worker bees with *M. plutonius* in the laboratory was associated with a significant decrease in survival in a dose-dependent and strain-dependent manner. Summer adult workers inoculated with 1 × 10^6^ 2019BC1 *M. plutonius* had 24.1% decrease in survival relative to control at experiment termination at day 24. By comparison, winter adult workers inoculated with 1 × 10^6^ 2019BC1 *M. plutonius* had 34.5% decrease in survival relative to control at experiment termination at day 30. The differences could be explained by the variances in physiology and age between summer and winter bees. Moreover, winter adult workers inoculated with 1 × 10^6^ ATCC *M. plutonius* had no significant difference in survival relative to control at experiment termination at day 30. The strain-associated differences in survival of adult bees inoculated with *M. plutonius* recapitulate the strain-associated differences in *in vitro* larval survival after *M. plutonius* infection, considering that CC13 strains such as the ATCC are considered nonpathogenic to honey bee larvae [4–6,8,21], while CC12 strains such as 2019BC1 cause high larval mortality *in vitro* [13]. Prior to this study, *M. plutonius* infection was only reported to cause mortality in newly hatched honey bee larvae [13]. Herein, we demonstrate that *M. plutonius* can also significantly reduce the survival of adult bees in the laboratory, although further study is needed to verify this finding in *M. plutonius*-colonized adult bees within a field colony.

We found that antibiotic treatment of adult workers in laboratory cages significantly improved survival of summer adult workers inoculated with *M. plutonius* compared to non-treated, *M. plutonius*-inoculated controls; however, antibiotic treatment did not eliminate *M. plutonius* colonization of adult workers maintained in the laboratory or in field colonies. Persistent detection of *M. plutonius* after antibiotic treatments in laboratory cages and in field colonies could be explained by the bacteriostatic nature of OTC [22] TYL and LMC [23,24], as bacteriostatic antibiotics only prevent the multiplication of bacteria, but do not kill the bacteria. Our study aligns with Masood et al. [13] where both approved (OTC) and non-approved (LMC and TYL) antibiotics for EFB were associated improved larval survival. Our results also align with the Budge et al. (2010) who reported that adult bees from colonies treated with oxytetracycline carried *M. plutonius,* but the bacterial load was significantly reduced [12]. We saw a decrease in bacterial load in both in the sham-treated and the antibiotic-treated groups and both sham-treated and antibiotic-treated bees had similar levels of *M. plutonius* detected in their body at experimental termination. Hence, this study does not show strong support for antibiotic efficacy in decreasing bacterial load, but it does support antibiotic efficacy in improving bee survival. Moreover, adult bees inoculated with *M. plutonius* and treated three times with OTC according to the label, or only one time with OTC, had similar survival in cage trials, but bacterial load post-treatment was higher in one-time treatment (5.1 × 10^4^ ± 7.1 × 10^4^) than three-times treatment (288.5 ± 221.5) at D15. This suggests that off-label, one-time dosing of colonies with OTC, may be similarly effective in improving survival of adult bees colonized with *M. plutonius*, although further field studies are necessary.

## 5. Conclusion

Our study showed that both summer and winter adult worker honey bees can remain persistently colonized with *M. plutonius*, and that colonization with a CC12 strain of *M. plutonius* is associated with significant decreases in adult bee survival in the laboratory. Therapy with both approved and non-approved antimicrobials for EFB was associated with improved survival of summer worker bees colonized with *M. plutonius*; however, adult bees remained persistently colonized with *M. plutonius*, despite antimicrobial therapy, highlighting the antibiotic treatment alone is ineffective for controlling EFB disease and that other IPM colony management strategies are necessary. These findings support adult honey bees as a reservoir of EFB disease and invite additional research focusing on the transfer of *M. plutonius* from adult bees to young larvae to elucidate the pathogenesis of this enigmatic disease.

## Supporting information

S1 Fig Mean (±SD) sugar consumption (µL/bee) of adult summer worker bees (n = 180/group) over time (in days) after inoculation with A. one of 6 incremental doses of of *M. plutonius* or B. one of 3 incremental (low, medium and high) doses of *M. plutonius* in laboratory cages. * and ** indicates significant differences from control with P < 0.05 and P < 0.01, respectively.
(PNG)

S2 Fig Mean (±SD) syrup consumption (µL/bee) of adult winter worker bees (n = 180/group) over time (in days) after inoculation with either *M. plutonius* ATCC 3511 or *M. plutonius* 2019BC1. * and ** Indicates significant differences from control with P < 0.05 and P < 0.01, respectively.
(PNG)

S3 Fig Mean (±SD) sugar consumption (µL/bee) of newly emerged summer worker bees in laboratory inoculated with 1 × 10^7^ CFU/bee *M. plutonius* 2019BC1 and or treated with oxytetracycline (OTC) lincomycin (LMC) or tylosin (TYL). “X” represents how many times treatment happed. '*' represents significant differences from D3 bacterial load, with P < 0.05.
(JPG)

S1 Table The bacterial count (CFU/mL) of inoculum provided to the bees on D1 (mean±SD). The sugar syrup inoculated with the *M. plutonius* was plated to KSBHI or basal agar plate to confirm the accuracy of the infectious dose administered on D1. Each cage contains 60 bees fed with 3 mL of *M. plutonius* inoculated sugar syrup.
(XLSX)

S2 Table *M. plutonius* culture on a pooled sample of 3 bees inoculated with *M. plutonius* in the laboratory. The samples from D3 and D22 were cultured on to KSBHI agar plates. The bacterial colonies grown in three replicate plates per sample were send to MALDI-ToF identification for *M. plutonius*.
(XLSX)

S3 Table *M. plutonius* culture on a pooled sample of 3 bees collected from field colonies. The samples from D3 and D22 were cultured on to KSBHI agar plates. The bacterial colonies grown in three replicate plates per sample were send to MALDI-ToF identification for *M. plutonius*.
(XLSX)

S4 Table *M. plutonius* culture on a pooled sample of 3 bees inoculated with *M. plutonius* in cages treated with one of the three antibiotics. The samples from D3 and D15/21 were cultured on to KSBHI agar plates. The bacterial colonies grown in three replicate plates per sample were send to MALDI-ToF identification for *M. plutonius*.
(XLSX)

S5 Table *M. plutonius* culture on a pooled sample of 3 bees from field colonies treated with OTC 3X. The samples from D3 and D15 were cultured on to KSBHI agar plates. The bacterial colonies grown in three replicate plates per sample were send to MALDI-ToF identification for *M. plutonius*.
(XLSX)

S6 Table *M. plutonius* culture on a pooled sample of 3 bees from laboratory cages inoculated with two strains of *M. plutonius.* The samples from D3 and D38 were cultured on to KSBHI agar plates. The bacterial colonies grown in three replicate plates per sample were send to MALDI-ToF identification for *M. plutonius*.
(XLSX)
