# Supplementary figures and images for: Antimicrobial control and temporal dynamics of M. plutonius colonization in adult worker honey bees (Apis mellifera)
Source: PLoS One. 2025 May 12;20(5):e0322770. doi: 10.1371/journal.pone.0322770 (PMC12068727; doi:10.1371/journal.pone.0322770)

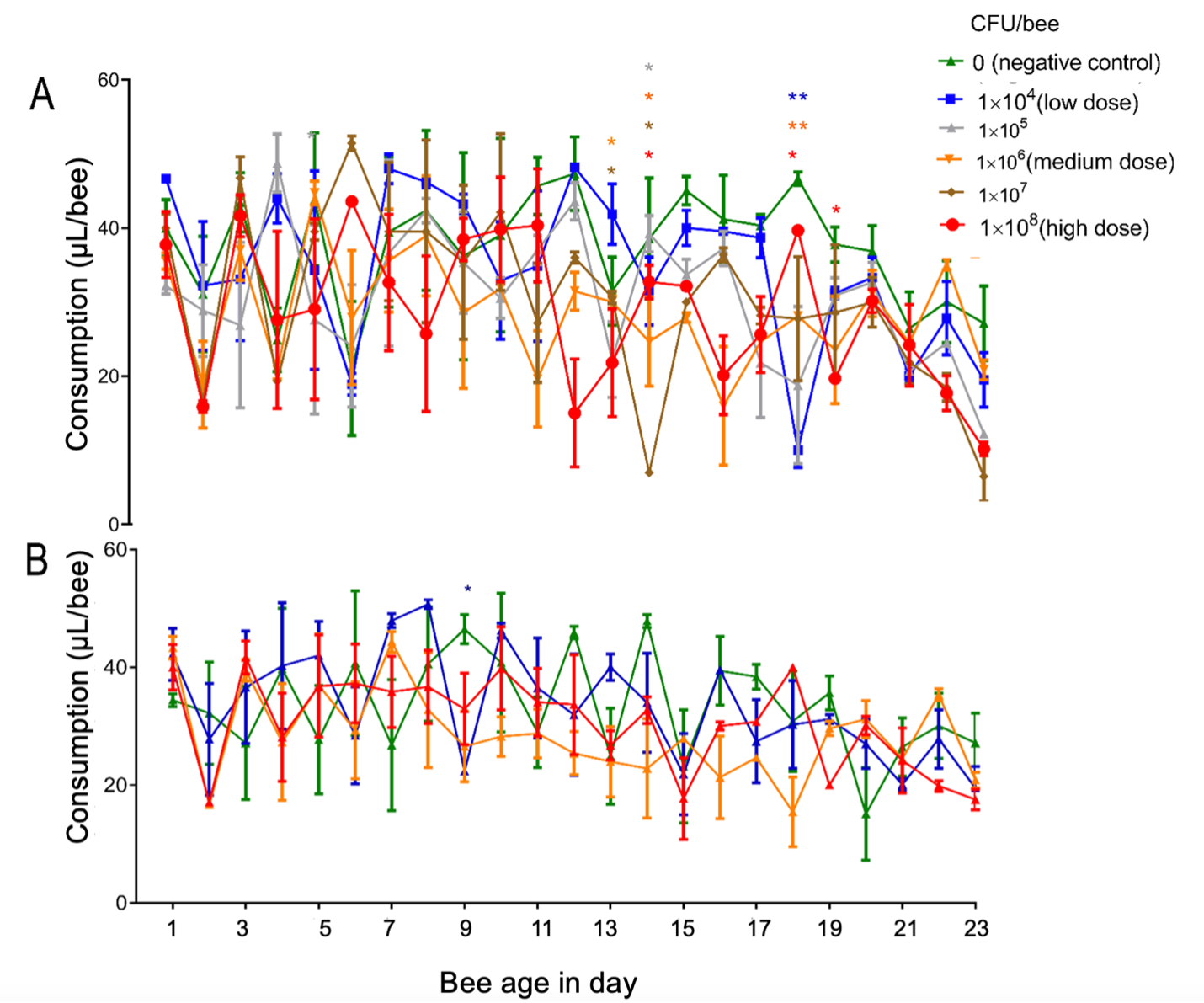

Supplement: S1 Fig — * and ** indicates significant differences from control with P < 0.05 and P < 0.01, respectively. (PNG) [file pone.0322770.s001.png]

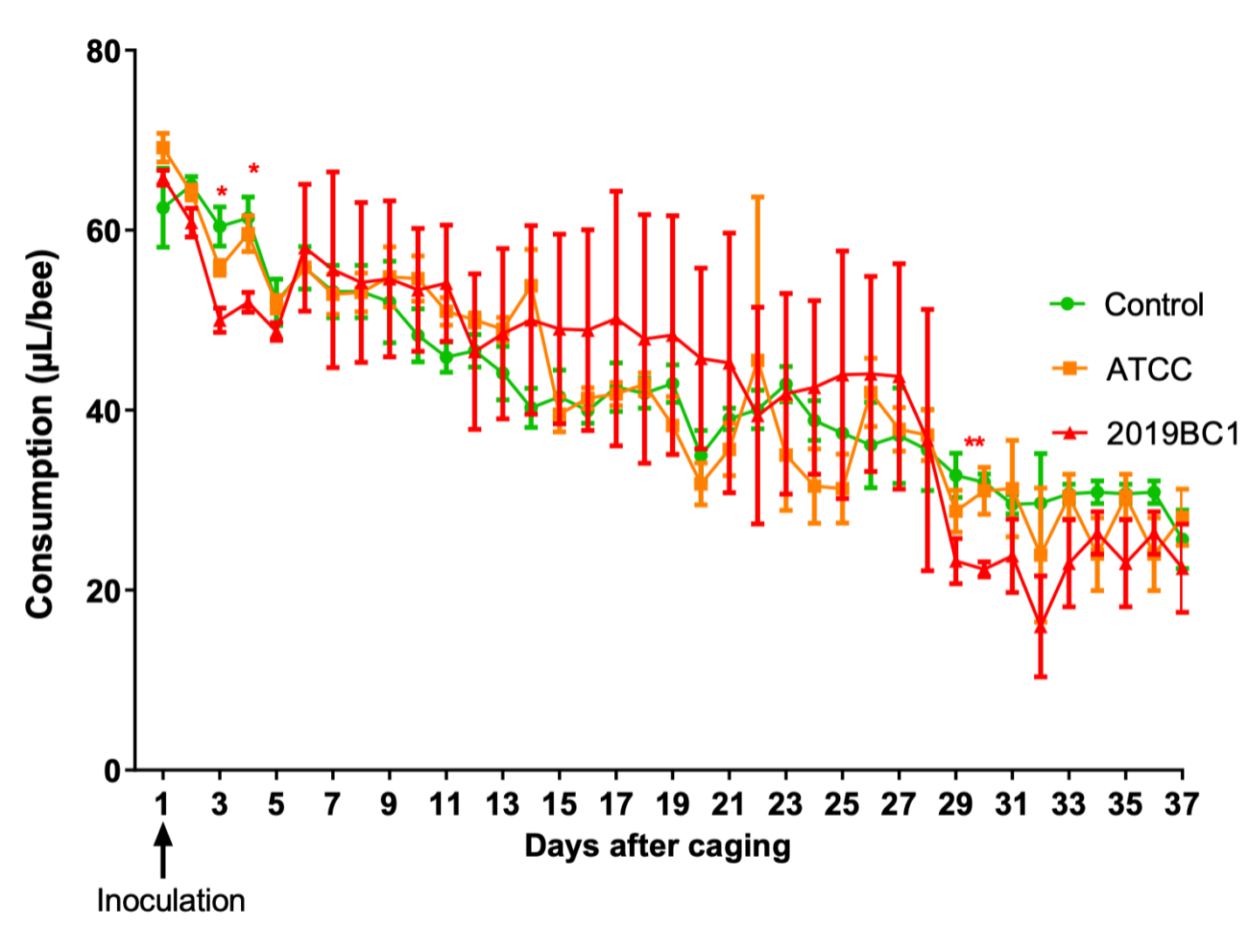

Supplement: S2 Fig — * and ** Indicates significant differences from control with P < 0.05 and P < 0.01, respectively. (PNG) [file pone.0322770.s002.png]

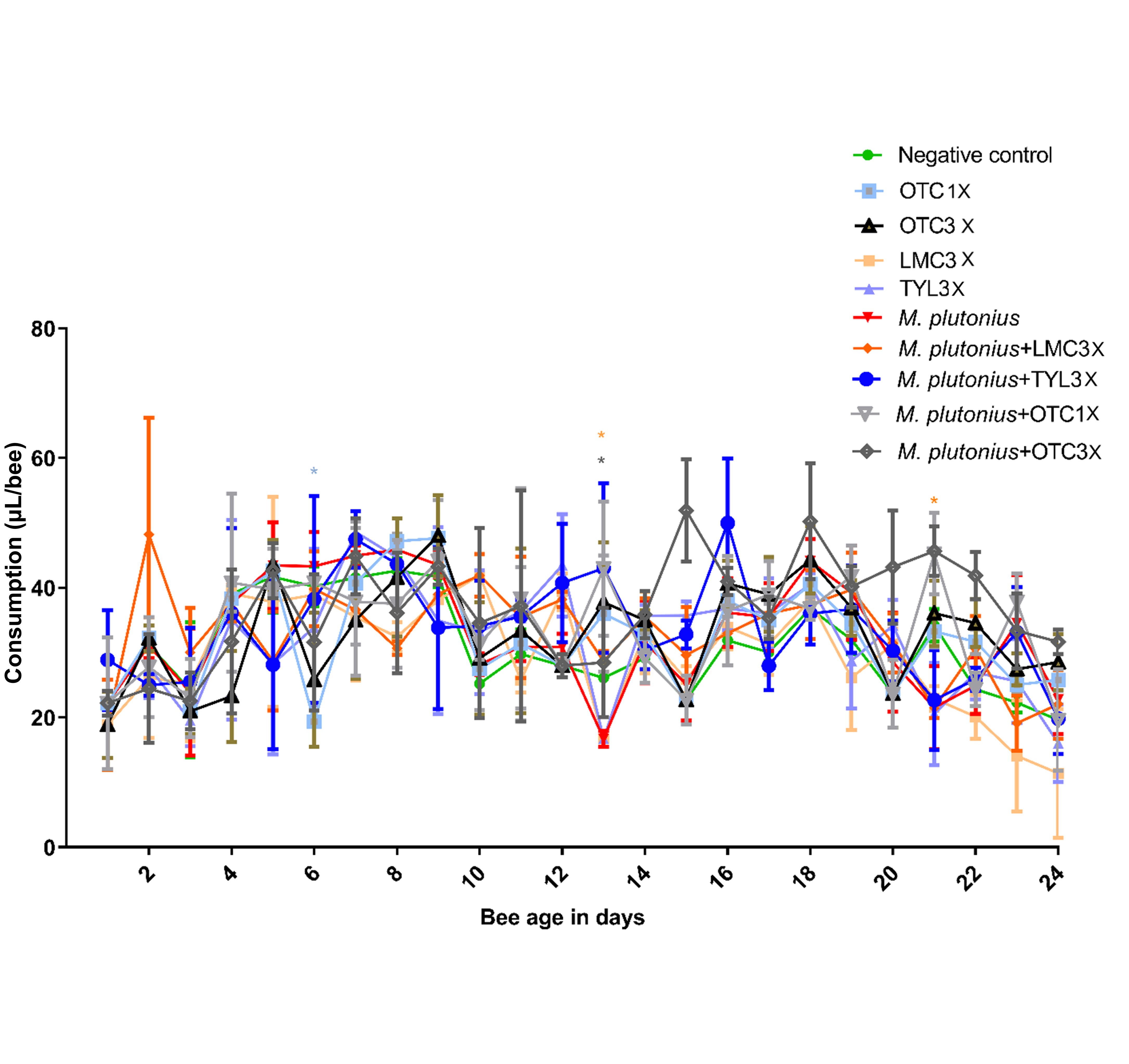

Supplement: S3 Fig — “X” represents how many times treatment happed. '*' represents significant differences from D3 bacterial load, with P < 0.05. (JPG) [file pone.0322770.s003.jpg]
